# Magnetic resonance imaging landmarks for preoperative localization of inferior medial genicular artery: a proof of concept analysis

**DOI:** 10.1186/s40634-020-00288-w

**Published:** 2020-09-28

**Authors:** Ennio Sinno, Armando Ugo Cavallo, Gianluca Cera, Michele Dell’Orfano, Daniele De Meo, Massimiliano Sperandio, Ciro Villani

**Affiliations:** 1 Department of Anatomical, Histological, Forensic Medicine and Orthopaedics Sciences, University “La Sapienza”, Piazzale Aldo Moro, 5, 00185 Rome, Italy; 2 Department of Biomedicine and Prevention, University “Tor Vergata”, Rome, Italy; 3 Division of Radiology, “San Carlo di Nancy Hospital”, GVM Care and Research, Rome, Italy

## Introduction

**Fig. 1:**
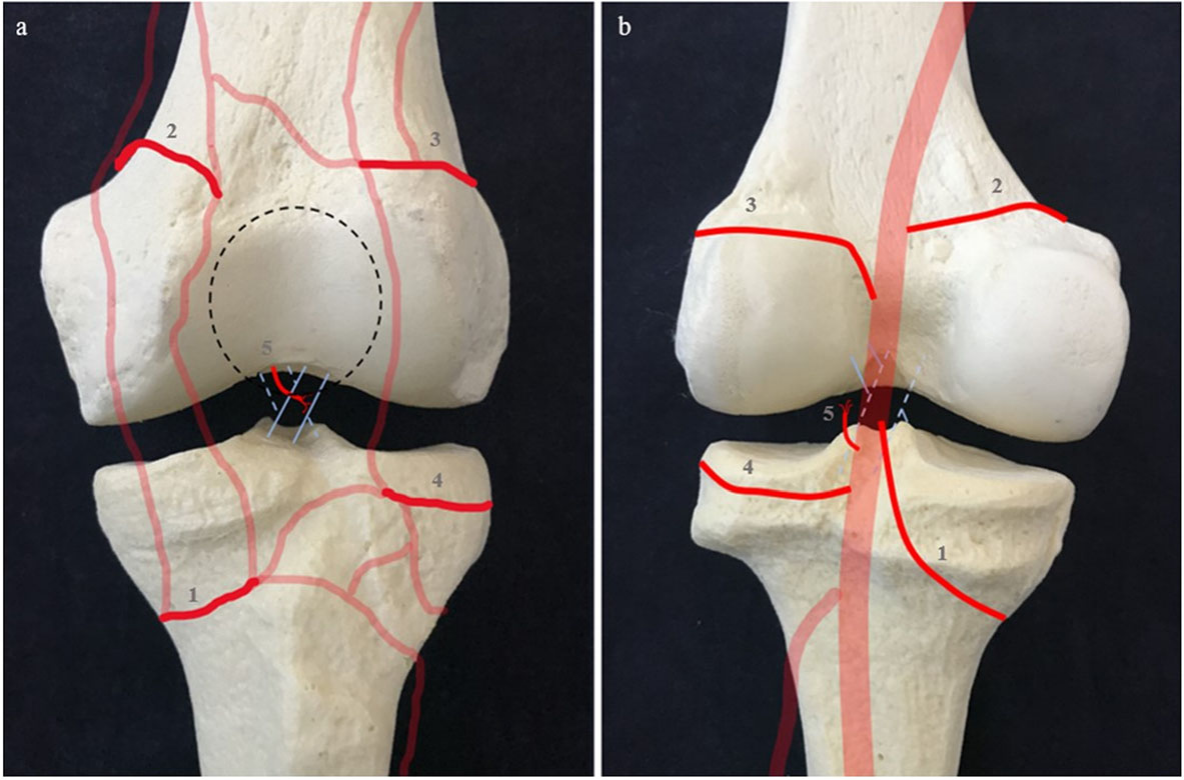
Anterior (**a**) and posterior (**b**) courses of arterial vessels surrounding knee joint (in red) drawn on a 3D model. Legend: 1.Inferior medial genicular artery. 2.Superior medial genicular artery. 3.Superior lateral genicular artery. 4.Inferior lateral genicular artery. 5.Middle genicular artery. *Black dotted line* Patella. *Light blue line* Anterior cruciate ligament. *Light blue dotted line* Posterior cruciate ligament

Popliteal artery (PA) provides the blood supply to the anatomical components of the knee. At the lower limit of the popliteal fossa PA divides into anterior and posterior tibial arteries. The superior medial genicular artery, superior lateral genicular artery, inferior medial genicular artery (IMGA), inferior lateral genicular artery (ILGA) and middle genicular artery (MGA) are all collateral branches arising from PA. The anastomotic complex deriving from these vessels in addition to the descending geniculate artery, and the anterior and posterior recurrent arteries of the anterior tibial artery, supplies both soft tissues surrounding the knee, articular and osseous structures (Fig. 1) [2, 10].

### Anatomy of IMGA

IMGA is one of the five collateral branches deriving from PA. Its origin can be superior, inferior or at the level of the joint line. From the popliteal fossa, the IMGA locates inferior to the medial condyle of the tibia, reaching the superficial medial collateral ligament [25]. Moreover, numerous anatomical variations had been reported in relation to its course. It could origin either directly as third, fourth or fifth branch from PA, or from a common trunk of PA, more frequently in combination with ILGA [23]. It supplies the medial proximal part of the tibia without any contribution from anastomotic vessels that supply the medial femoral condyle [12, 20, 27]. Then, it moves anteriorly to form an anastomotic complex with ILGA in the infrapatellar fat pad, thus supplying the patella [11]. On its course, it provides cutaneous branches perforating at the lateral border of the sartorius muscle and at the medial border of the quadriceps tendon. Finally, it forms a common trunk with ILGA that initially branches to supply the anterior cruciate ligament (ACL) and then travels through the intraarticular space to form a peri ligamentous anastomosis with the MGA, thus giving bloody supply to the postero-superior cruciate zone [17].

Numerous complications following its lesion after a variety of knee surgery procedures had been reported in the literature [3, 4, 6–9, 14–16, 18, 22, 24].

Standard approaches that involve the medial aspect either of knee joint or of the tibial metaphysis, could determine a lesion to this vessel probably due to its numerous interindividual anatomical variations. On this basis, we retrospectively reviewed the anatomical course of IMGA on magnetic resonance imaging (MRI) scans in order to identify reproducible landmarks, which could be useful for preoperative planning and surgical procedures, that could help orthopedic surgeons to identify this vessel, thus to avoid any iatrogenic lesion.

## Methods

### Patients

Magnetic Resonance images of the knee obtained with a high magnetic field MR scanner (Aera, 1.5 T, Siemens, Erlangen, Germany) were evaluated. Patients that underwent knee MRI due to unspecific knee pain were enrolled. Eighty MRI from 80 patients (34 males and 46 females) were considered. The age of all patients was 50.7 ± 15 years (44.8 ± 17.6 in males, 55 ± 11.1 in females). All knee MRIs evaluated have been performed with standard protocols without gadolinium injection. Inclusion criteria were no previous knee and lower limb vascular surgeries, arteriopathies and absence of arthritis deformity or direct trauma. Exclusion criteria were history of diabetes and smoking habits. Written informed consent was obtained by all patient.

### MRI technique and measurements

The measurements were conducted on T2 weighted (T2w) with fat suppression sagittal (TR: 960 ms; TE 23 ms; slice thickness 4 mm) and coronal (TR: 3650 ms; TE 38 ms; TI 160 ms; slice thickness 4 mm) sequences. The MRI scan was conducted in supine position, with the knee in extension and the lower limb in neutral rotation.

**Fig. 2:**
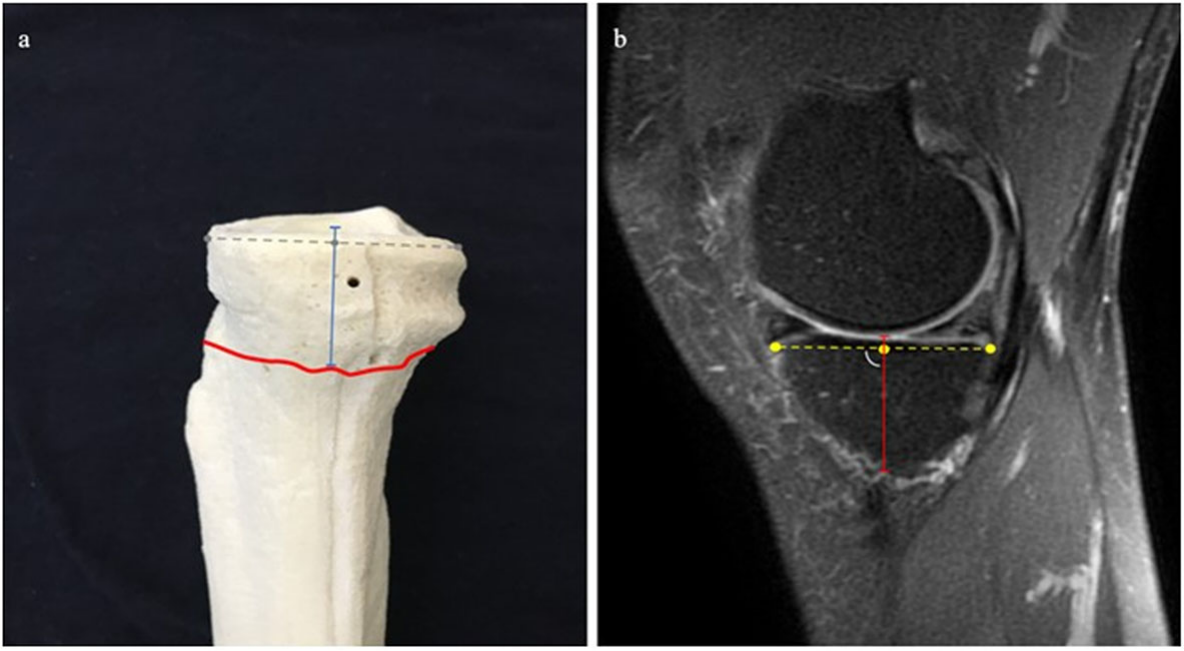
**a** IMGA–JL Sag (blue) drawn on a 3D model. IMGA course in red and the reference line of the medial tibial plateau in grey. **b**. IMGA-JL Sag on T2w with fat suppression sagittal sequence (red)

**Fig. 3:**
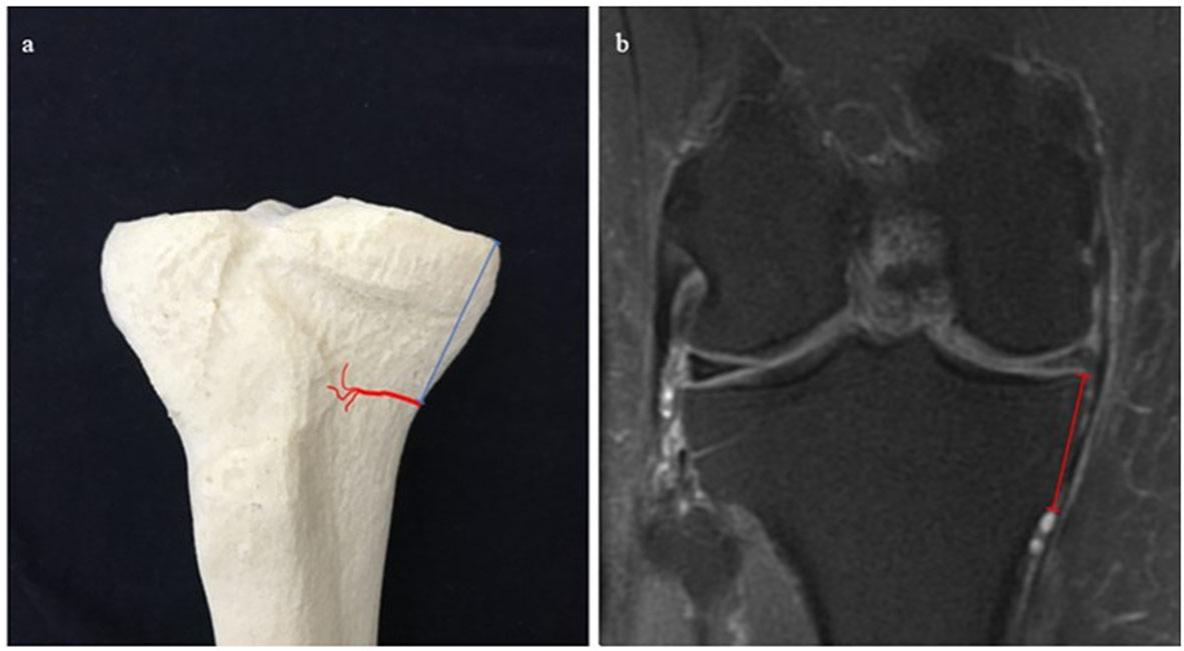
**a** IMGA–JL Cor (blue) drawn on a 3D model. IMGA course in red and the reference line of the anterior tibial plateau in grey. **b**. IMGA-JL Cor on T2w with fat suppression coronal sequence (red)

**Fig. 4:**
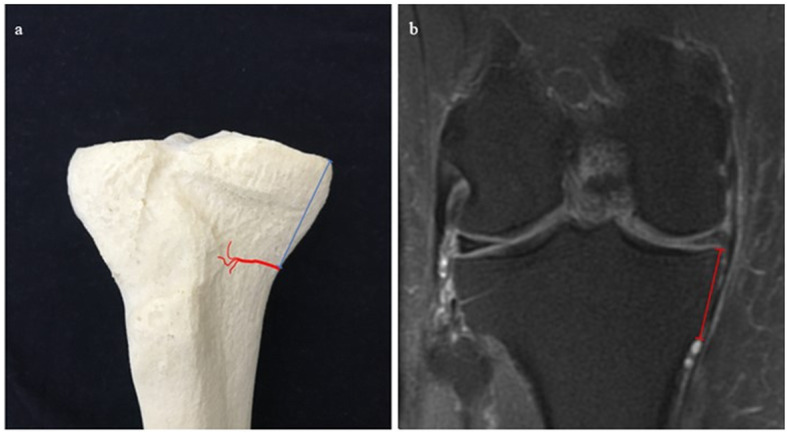
**a** IMGA–MTP Cor (blue) drawn on a 3D model. IMGA course in red. **b**. IMGA-MTP Cor on T2w with fat suppression coronal sequence (red)

**Fig. 5:**
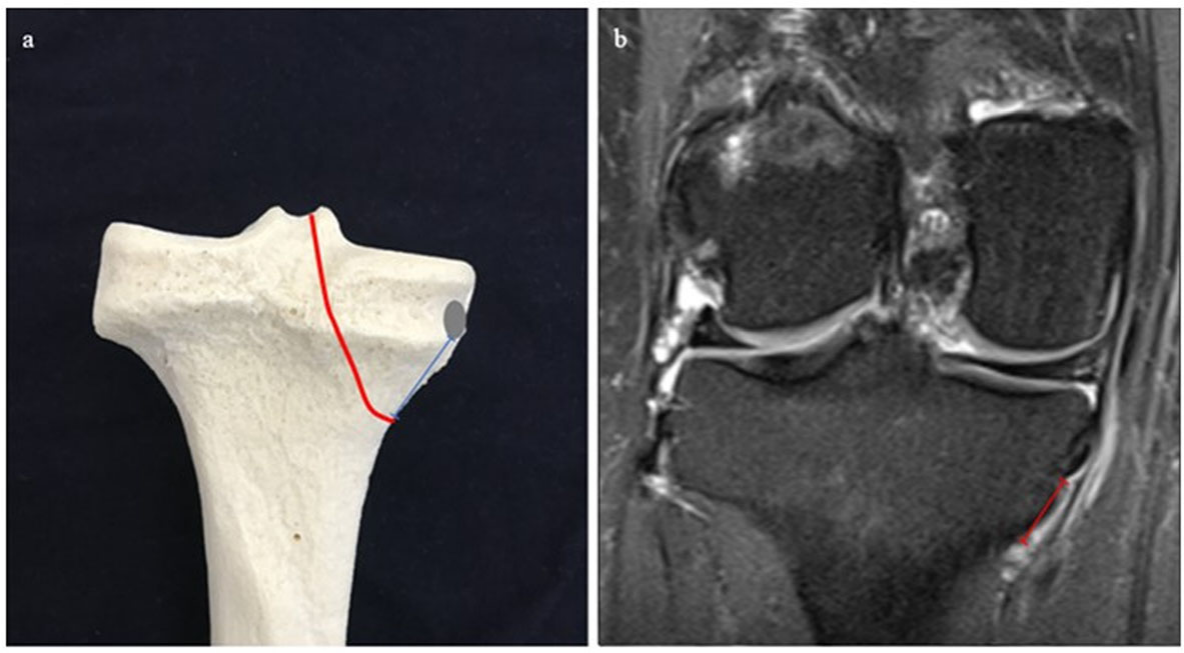
**a** IMGA–SMT Cor (blue) drawn on a 3D model. IMGA course in red. Semimembranous tendon in grey. **b**. IMGA-SMT Cor on T2w with fat suppression coronal sequence (red)

Four parameters were considered for final analysis:

- distance from IMGA to the joint line in the sagittal view (IMGA-JL Sag) (Fig. 2),
- distance from IMGA to the joint line in the coronal view (IMGA-JL Cor) (Fig. 3),
- distance from IMGA to the border of the medial tibial plateau in the coronal view (IMGA-MTP Cor) (Fig. 4) and
- distance from IMGA to the insertion of semimembranous tendon on the posterior part of the medial tibial plateau in the coronal view (IMGA-SMT Cor) (Fig. 5).

The average value (IMGA-JL Avg) between IMGA-JL Sag and IMGA-JL Cor was also calculated.

All the measurements were performed twice by two orthopedic surgeons with a temporal interval of 6 weeks.

For sake of standardization a technical procedure was applied to each MRI scan: first, the identification of IMGA on T2w sagittal and coronal sequences was evaluated; then, in T2w sagittal sequence, a line dividing the medial tibial plateau into two equal parts was drawn and the distance passing perpendicularly to that line, from IMGA to joint line (IMGA-JL Sag) was measured.

Then, on T2w coronal sequence, a line was drawn from the medial to the lateral border of the tibial plateau and the distance from IMGA to joint line (IMGA-JL Cor), was measured.

In the same sequence the distance from IMGA to the bony border of the tibial medial plateau (IMGA-JL Cor) was calculated. Finally, on T2w coronal sequence, the insertion of the semimembranosus tendon was identified on the posterior aspect of the medial tibial plateau and the distance from IMGA to it (IMGA-SMT Cor) was calculated.

### Statistical analysis

Statistical analysis was performed with R V 3.4.4 (R Core Team (2018). R: A language and environment for statistical computing. R Foundation for Statistical Computing, Wien, Austria. URL https://www.r-project.org/). Continuous variables were analyzed with t-test or Wilcoxon test when appropriate. Inter- and intra-observer agreement was tested using the intraclass correlation coefficient (ICC). A *p*-value < 0.05 was considered statistically significant.

## Results

Considering all cases, IMGA-JL Sag was 30 ± 4.3 mm, IMGA-JL Cor was 29.9 ± 4 mm, IMGA-JL Avg was 29.9 ± 4 mm, IMGA-MTP Cor was 30 ± 4.2 mm and IMGA-SMT Cor was 8.3 ± 2.1 mm (Table 1). There was no statistically significant difference between IMGA-JL Sag and IMGA-JL Cor (*p* = 0.9). In males the value of IMGA-JL Sag was 32.7 ± 3.8 mm, in females was 28 ± 3.5 mm (*p* < 0.001); the value of IMGA-JL Cor was 32.5 ± 3.2 mm in males, 27.9 ± 3.3 mm in females (*p* < 0.001). IMGA-JL Avg was 32.6 ± 3.3 mm in males, 27.9 ± 3.2 mm in females (*p* < 0.001). IMGA-MTP Cor was 32.8 ± 3.4 mm in males, 28 ± 3.4 mm in females (*p* < 0.001). IMGA-SMT Cor was 8.7 ± 2 mm in males, 8 ± 2.2 in females respectively (*p* = 0.1) (Table 2). The intraobserver agreement was 0.98 and interobserver agreement was 0.99 for the first measurement and 0.98 for the second measurement performed by both observers after 6 weeks.

**Table 1:** Baseline characteristics of patients included in the analysis

Age	50.7 ± 15
IMGA-JL Sag (mm)	30 ± 4.3
IMGA-JL Cor (mm)	29.9 ± 4
IMGA- JL Avg (mm)	29.9 ± 4
IMGA-MTP Cor (mm)	30 ± 4.2
IMGA-SMT Cor (mm)	8.3 ± 2.1

**Table 2:** Comparison between male and female patients

	M	F	***p***-value
Age	44.85 ± 17.57	55 ± 11.11	0.005
IMGA-JL Sag (mm)	32.66 ± 3.8	28.04 ± 3.49	< 0.001
IMGA-JL Cor (mm)	32.57 ± 3.23	27.86 ± 3.35	< 0.001
IMGA-JL Avg (mm)	32.61 ± 3.32	27.95 ± 3.18	< 0.001
IMGA-MTP Cor (mm)	32.83 ± 3.43	27.98 ± 3.45	< 0.001
IMGA-SMT Cor (mm)	8.75 ± 2.02	7.96 ± 2.18	0.1

## Discussion

The most important finding in this study is that using MRI scans, routinely performed as preoperative instrumental investigation before knee surgery, it is possible to identify reliable reference points useful to avoid damage to IMGA during knee surgery. Furthermore it seems easy to recognize IMGA on MRI T2w with fat suppression sequences. With this approach, no further investigation, such as angio-CT or angiography could be needed to this purpose, resulting in cost-effectiveness for the patient. Moreover, these instrumental examinations, imply the infusion of contrast agents and contrast-specific techniques all having potential side effects as well as relative and absolute contraindications (allergic reaction, caution in case of patient affected by renal insufficiency, etc).

The correct application of the calculations shown above allows the localization of the IMGA course with reference to the joint line and the medial edge of the tibial plateau. The calculation of this distance allows the surgeon to act safely, avoiding lesions of this vessel. Using MRI it is also possible to measure the distance between IMGA and the tendon of the semimebranous, an anatomically important element in knee prosthetic surgery.

There are few studies that analyze the anatomic course of IMGA, either cadaveric or angiographic [2, 5, 10, 17, 23].

To the best of our knowledge, it exists only one study based on MRI findings in relation to ILGA course [19]. Then, considering the risk of iatrogenic lesion of IMGA, it was attempted to assess its localization on MRI scans. All the measurements reported can be easily reproduced, as illustrated in the figures (Figs. 2, 3, 4 and 5). A useful landmark for surgical approaches to knee is the joint line. On this basis, the assessment of the distance between joint line and IMGA course could represent a valid guide for surgeons to avoid iatrogenic damage to this vessel. Being closely to real anatomy, this distance (IMGA-JL Avg) represents the mean value of the two measurements (IMGA-JL Sag and IMGA-JL Cor) drawn on MRI scans (Figs. 2 and 3). It was observed that IMGA-JL Avg had an interindividual variability, but considering the total amount of the male population, this length resulted greater (32.6 ± 3.3 mm) than female (27.9 ± 3.1 mm) (*p* < 0.001). This discrepancy could be associated to the well-known different physiognomic features between males and females. As a result, IMGA course could be closer to the joint line in females than in males, resulting in obvious surgical implications. Another measured distance (IMGA-MTP Cor) was drawn from IMGA to the border of tibial medial plateau: this bony point was chosen in order to provide a reproducible and useful landmark for surgeons. Finally, it was considered the distance between IMGA and the tibial insertion of the semimembranous tendon (IMGA-SMT Cor). Although extremely variable between patients, this distance could reveal a consistent practical usefulness for surgeons, specifically during TKA procedures. Whiteside described surgical tecnique for ligament balancing for TKA and focusing on medial compartment, he suggested that, in specific cases, it was necessary to extend the release up to the contracted semimembranous [26]. In these cases, surgeon should proceed with caution to preserve the integrity of IMGA. In our population the mean value of IMGA-SMT Cor was 8.2 ± 2.1 mm, suggesting a not negligible relation between these structures, although there were not observed statistically significant difference in males and females.

All knee surgical procedures could cause various iatrogenic injuries of this vessel. Nevertheless, there are several papers reporting post-surgical complications due to IMGA lesion, the most frequent of which seems to be pseudoaneurysm [3, 6–9, 16, 18, 22, 24]. Indeed, Filho et al. reviewed 40 papers in literature obtaining that the arteries most affected were the PA (45.83%) and the IMGA (20.83%) [9]. Patients affected by pseudoaneurysm referred to clinicians mainly for pain (36.55%) and pulsatile tumor (31.18%).

Medial approaches to proximal tibia could determine an IMGA lesion, due to its relationship with anatomic structures of the medial compartment of the knee.

Focusing on IMGA injury after arthroscopic procedures, partial medial meniscectomy had been reported as a possible cause [7]. It is well known to knee surgeons that the resection of the posterior horn of the medial meniscus should be performed avoiding excessive tibial external rotation. This maneuver brings closer PA and IMGA to this medial meniscus part [4]. Moreover, solely standard medial arthroscopic portal could lead to IMGA lesion, regardless the procedure performed [14].

ACL surgery could cause IMGA injury. Evans et al. reported the first case of IMGA pseudoaneurysm following ACL reconstruction using bone patellar tendon bone autograft; vessel damage was probably caused by the surgical technique, particularly while performing the tibial tunnel when the tibial periosteum needs to be lifted [8]. Mello et al., reporting another case of IMGA pseudoaneurysm after ACL reconstruction using bone patellar tendon bone autograft, hypothesized that the damage might be due to the preparation of the intercondilar notch, performed to have a good view of its roof and posterior wall. This procedure usually provides the removal of the posterior cruciate ligament synovial membrane which is fed by IMGA [15]. Moreover, Milankov et al. described IMGA injury after ACL reconstruction using quadrupled hamstring tendon. In this case the reason of the vascular complication could had been the graft removal from the medial side of proximal tibia [16].

In ACL reconstruction surgery using hamstring tendon, IMGA lesion could compromise the success of the procedure as its terminal branches may influence the maturation of the graft [1].

Even during high tibial osteotomy (HTO) procedures IMGA is at risk of injury; Bisicchia et al. tried to assess the risk of vascular injuries both in closing and opening wedge HTOs, quantifying the distances between osteotomy cuts and arteries, using three-dimensional reconstruction: it was observed that IMGA could be more at risk during opening wedge HTOs [5].

Unicompartmental knee arthroplasty or TKA are more commonly performed through medial arthrotomy nearly the IMGA course; a considerable bleeding may occur during surgery, from inferomedial side of the joint capsule probably caused by IMGA injury. Cauterization of the soft tissues on this side is often necessary to allow surgeons to have a proper view of the area. Some authors reported their experience on IMGA pseudoaneurysm after TKA, suggesting a possible injury mechanism caused by the release of medial structures during ligament balancing techniques in patients suffering for knee osteoarthritis associated with varus deformity [26]. During this procedure, care must be taken by the surgeon to avoid any damage to IMGA, since its anatomical strictly connection with MCL, proximal tibial periosteum, pes anserinus insertion, posterior oblique ligament and semimembranous tendon [6, 18].

Finally, trauma surgery for tibial fractures may be involved in IMGA lesion too. Bennet et al. reported a case of IMGA false aneurysm after tibial intramedullary nailing revision surgery [3]. Either the removal of the prior proximal interlocking screw from the medial side of proximal tibia or the reinsertion of the new one, could have caused the lesion. Thus, the surgical treatment of fractures involving the medial tibial plateau, best managed by subperiosteal internal fixation, could endanger IMGA. However, reports of pseudoaneurysm formation without direct vessel wall injury have also been described [9, 13, 21].

This study tries to emphasize the importance of IMGA and its collateral branches supply to the medial compartment of the knee. For this reason, a lesion of this vessel could determine surgical failures or insufficient view of the surgical area. Providing accurate landmarks to identify IMGA course in the medial compartment could represent a useful tool for surgeons in order to avoid its iatrogenic lesions.

Furthermore, to our knowledge there are no similar investigations in literature referring to MRI evaluation of IMGA course.

Several limitations can be listed:

- the measurements were conducted only on MRI scans, without any in vivo confirmation;
- the determination of accurate references was made by the Authors on the basis of anatomical landmarks detected on MRI scans;
- all patients enrolled in the study came from the same ethnic group. Because of the difference in anatomical features between groups, caution in required in applying our results to general population;
- the evaluations were conducted on a small sample of MRIs. An analysis based on a more consistent group would increase the statistical accuracy of the measurements.

## Conclusion

The joint line is a useful landmark to identify IMGA course during knee surgery. The IMGA course is closer to the joint line and to the border of the medial tibial plateau in females than in males. Although the interindividual variability these results should be taken into account when performing all surgical procedures involving the medial aspect of the knee. Similar interindividual distances were observed between IMGA and semimembranosus tendon insertion regardless of gender. However, the proximity to this tendon should be considered especially during specific cases of ligamentous balancing in TKA procedures.
